# Isolation of Elusive HAsAsH in a Crystalline Diuranium(IV) Complex

**DOI:** 10.1002/anie.201508600

**Published:** 2015-10-28

**Authors:** Benedict M Gardner, Gábor Balázs, Manfred Scheer, Ashley J Wooles, Floriana Tuna, Eric J L McInnes, Jonathan McMaster, William Lewis, Alexander J Blake, Stephen T Liddle

**Affiliations:** School of Chemistry, University of Manchester Oxford Road, Manchester, M13 9PL (UK) E-mail: steve.liddle@manchester.ac.uk; Institut of Inorganic Chemistry, University of Regensburg, Universitätsstrasse 31 93053 Regensburg (Germany) E-mail: manfred.scheer@ur.de; School of Chemistry, University of Nottingham, University Park Nottingham, NG7 2RD (UK)

**Keywords:** arsenic, back-bonding, density functional theory, diarsene, uranium

## Abstract

The HAsAsH molecule has hitherto only been proposed tentatively as a short-lived species generated in electrochemical or microwave-plasma experiments. After two centuries of inconclusive or disproven claims of HAsAsH formation in the condensed phase, we report the isolation and structural authentication of HAsAsH in the diuranium(IV) complex [{U(Tren^TIPS^)}_2_(μ-η^2^:η^2^-As_2_H_2_)] (**3**, Tren^TIPS^=N(CH_2_CH_2_NS*i*Pr^i^_3_)_3_; Pr^i^=CH(CH_3_)_2_). Complex **3** was prepared by deprotonation and oxidative homocoupling of an arsenide precursor. Characterization and computational data are consistent with back-bonding-type interactions from uranium to the HAsAsH π*-orbital. This experimentally confirms the theoretically predicted excellent π-acceptor character of HAsAsH, and is tantamount to full reduction to the diarsane-1,2-diide form.

Dipnictenes REER (E=N, P, As, Sb, Bi; R=H, alkyl, aryl) are a fundamental class of molecules that have played a central role in the development of main-group chemistry.[1] Diazenes have been known for decades and are most prevalent, and though diphosphenes, diarsenes, distibenes, and dibismuthenes have all been reported in the past thirty years, their numbers rapidly decrease down the group.[1] This reflects the difficulties of stabilizing multiple bonds between increasingly large nuclei, the importance of dispersion forces,[1a] and the reduced tendency of heavier p-block elements to catenate, and thus sterically demanding substituents are required to stabilize these linkages.[1] However, it is fundamentally appealing to study parent REER molecules (R=H), free of structural distortions caused by bulky stabilizing groups, to more clearly probe their potential π-acceptor properties when bonded to metal centers. However, the combination of a double bond and lone pairs renders dipnictenes increasingly reactive; HNNH is only found in the solid state when coordinated to metals,[2, 3] and only three metal–HPPH complexes are known.[4] Conspicuously, there are no examples of structurally authenticated HEEH (E=As, Sb, and Bi) in any charge state and therefore little is known about these parent molecules.

Where HAsAsH is concerned, generation in electrochemical–IR and microwave plasma–IR experiments has been proposed,[5] but the assignments, whilst consistent with As–H bonds, were not conclusive regarding the precise nature of these transient, surface-absorbed hydrides. In the routine condensed phase, HAsAsH was first proposed as a reaction product in 1810 by Davy[6] and a year later by Gay-Lussac and Thénard.[7] In 1924, Weeks and Druce claimed that the action of stannous chloride on arsenic trichloride in the presence of hydrochloric acid produced brown, amorphous solids formulated as HAsAsH.[8] However, in 1957, Jolly, Anderson, and Beltrami showed that these products are ostensibly arsenic with adsorbed sub-stoichiometric arsenic hydrides.[9] Thus, HAsAsH has eluded capture, and has most likely never actually been made, which probably reflects the absence of synthetic methods to construct HAsAsH and prevent subsequent decomposition in the absence of bulky arsenic-bound stabilizing groups. Here, more than two centuries after it was first proposed, we report the synthesis and structural authentication of HAsAsH in a crystalline diuranium(IV) complex.

We previously reported that the [U(Tren^TIPS^)] (Tren^TIPS^=N(CH_2_CH_2_NSiPr^i^_3_)_3_) fragment stabilizes reactive fragments such as *cyclo*-P_5_,[10] mono-oxo,[11] terminal nitrides (U≡N),[12] and parent U=EH groups (E=N, P, As).[13–15] The latter of these was prepared by reaction of [U(Tren^TIPS^)(THF)][BPh_4_] (**1**)[14] with KAsH_2_,[16] to give [U(Tren^TIPS^)(AsH_2_)] (**2**)[15] followed by deprotonation of **2** and abstraction of the potassium cation by a crown ether. Inspired by the works of Herrmann and Huttner,[17] we wondered whether **2** could undergo oxidative homocoupling to give HAsAsH stabilized by a bulky [U(Tren^TIPS^)] unit that might preclude decomposition.

To prepare **2**, the KAsH_2_ reagent has to be finely ground, otherwise intractable product mixtures are obtained.[15] However, on one occasion a small crop of dark brown crystals of [{U(Tren^TIPS^)}_2_(μ-η^2^:η^2^-As_2_H_2_)] (**3**) was obtained in about 1 % yield. Deducing that **3** is likely formed due to sluggish KAsH_2_ reactivity when not ground, therefore resulting in localized excesses of KAsH_2_ deprotonating **2** when formed, we repeated the reaction with different ratios of **1**:KAsH_2_ varying from 1:1.1 to 1:2 where KAsH_2_ was ground. We determined 1:1.4 to be the optimal ratio, which reproducibly affords **3** in circa 50 % crude yield, as determined by ^1^H NMR spectroscopy using 2,4,6-Bu^t^_3_C_6_H_3_ as an internal standard (Scheme 1).[18] Recrystallization reproducibly affords **2** in 8 % pure crystalline yield, reflecting the instability of HAsAsH.

**Scheme 1:**
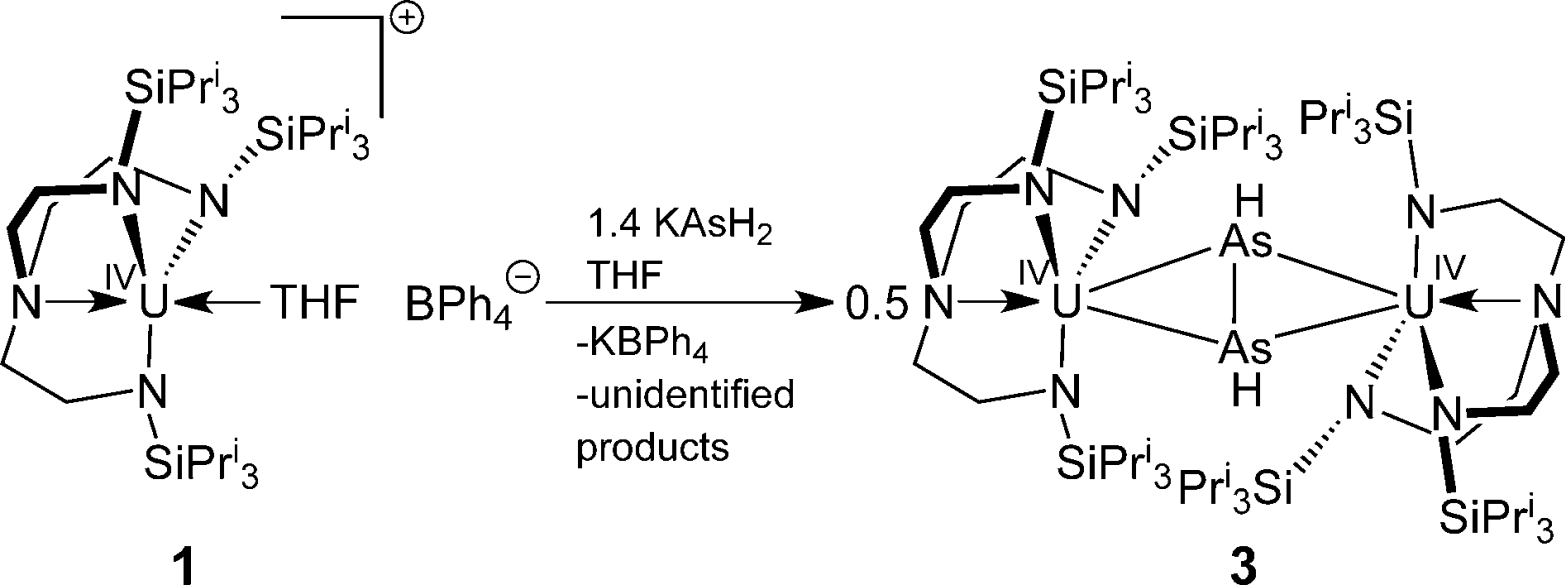
Synthesis of 3 from 1 and KAsH_2_.

Whilst it seems certain that the KAsH_2_ deprotonates **2** to give [U(Tren^TIPS^)(AsHK)], and presumably AsH_3_, in situ, it is unclear how it promotes oxidative homocoupling to give **3**, as all attempts to identify by-products have been inconclusive. However, we note that AsH_3_ has precedent for forming MAsH_2_ and H_2_ (M=Na or K) from M-containing substrates, and that KAsH_2_ is known to decompose to “KAs” and H_2_, which might provide the redox path to **3**.[16] We also note that although **3** is obtained most conveniently by treatment of **1** with excess KAsH_2_, rather than isolating **2** and reacting with further KAsH_2_, the latter method is effective, suggesting that the HAsAsH unit may be formed by coupling and subsequently remains isolated between two uranium centers. We investigated alternative methods of producing **3**, by preparing [U(Tren^TIPS^)(AsHK)] and treating it with oxidants to effect homocoupling; however, adding stoichiometric iodine, lead(II) iodide, TEMPO, pyridine-*N*-oxide, 4-morpholine-*N*-oxide, trimethylamine-*N*-oxide, silver tetraphenylborate, and copper(I) iodide all gave intractable products. We have also separately refluxed and photolyzed **2** to see if dihydrogen elimination to give **3** occurs, but only decomposition occurs under these conditions. These observations underscore the fragile nature of HAsAsH and hence why it was elusive. Although **3** is obtained in poor crystalline yield or moderate yield in crude form, the synthesis is reproducible.

On one occasion, after isolating **3**, a small crop of light brown crystals deposited from the mother liquor in less than 1 % yield. These were identified as [{U(Tren^TIPS^)}_2_(μ-η^2^:η^2^-As_2_)] (**4**).[18] Although the low yield of **4** has prevented its characterization, its structure serves to support the formulation of **3** by virtue of their metrical differences, and underscores the complex dehydrogenative chemistry that operates for these redox active molecules with polar bonds.[16]

Once **3** is crystalline, it has very low solubility in non-polar solvents and it decomposes in polar solvents, so reliable UV/Vis/NIR spectra could not be obtained. The ^1^H NMR spectrum exhibits two very broad resonances at circa 5.3 (Pr^i^) and circa 6.2 ppm (CH_2_); we attribute this to the dinuclear nature of **3** and the absence of As–H resonances to their close proximity to the paramagnetic uranium ions. The ATR-IR spectrum of **3** exhibits one weak As–H absorption at 2029 cm^−1^ (2052 and 2031 cm^−1^ for **2**),[15] and this compares to As–H absorbances at 2040 and 2000 cm^−1^ assigned as HAsAsH generated in situ deposited on GaAs surfaces,[5a],[b] but is significantly different to As–H stretches of 2306 and 2298 calculated for gas-phase HAsAsH.[19] An analytical frequency calculation predicts symmetric and asymmetric As–H stretches in the IR spectrum of **3** at 2049 and 2028 cm^−1^, respectively, which for the latter compares well to the experimentally observed value. The As–H stretch at 2029 cm^−1^ can thus be assigned as the asymmetric stretching mode, because due to selection rules the symmetric stretch cannot be IR active as **3** exhibits an inversion center (see below). The symmetric stretch should be observable in the Raman spectrum of **3**, but samples of **3** decompose in the beam, or the inherently weak As–H stretch cannot be observed at low-/mid-power settings or in dilute samples, so this data remains unobtainable. The ATR-IR data for **3** rule out the presence of the *Z* isomer because, lacking an inversion center, it would exhibit both symmetric and asymmetric As–H stretches, which is not observed experimentally. To examine this aspect further we prepared [{U(Tren^TIPS^)}_2_(μ-η^2^:η^2^-As_2_D_2_)] (**3 D**), using previously unknown KAsD_2_.[18] As anticipated the ATR-IR spectrum of **3 D** does not exhibit the absorbance at 2029 cm^−1^, but the As–D stretch could not be observed because from reduced-mass considerations this absorbance falls in the fingerprint region where a strong and broad absorbance (1410–1490 cm^−1^) resides.

The molecular structure of **3** is shown in Figure 1;[18] the salient feature is the presence of HAsAsH bridging two [U(Tren^TIPS^)] units. In the solid state, **3** crystallizes over an inversion center between the two arsenic ions. Although this is consistent with the presence of the *E* isomer, in the crystal that was examined the hydride is disordered over two sites, so the presence of the *Z* isomer, as opposed to two averaged *E* isomers of opposite “hands”, could not initially be discounted. However, the ATR-IR data rule out the presence of the *Z* isomer, which is consistent with the greater prevalence of *E* dipnictenes compared to the corresponding *Z* isomers.[1, 20] The uranium–amide and uranium–amine bond lengths in **3** are typical of such distances.[21] The U–As distances of 3.1203(7) and 3.1273(7) Å in **3** are longer than the sum of the single bond covalent radii for uranium and arsenic (2.91 Å),[22] but are only slightly longer than the formal U–As covalent σ-bond in **2** (3.004(4) Å).[15] The As–As bond length in **3** of 2.4102(13) Å is consistent with a single rather than double bond,[23] the latter of which tends to be about 2.2 Å,[1] and rules out the presence of an (As_2_) unit that when trapped between two transition metals exhibits As–As bond lengths of circa 2.2–2.3 Å (see **4** below).[17, 23a, 24] When diarsenes with sterically demanding substituents are bonded to transition metals the As–As bond tends to lengthen as a result of back-bonding, for example to 2.365 Å in [(CO)_4_Fe(η^2^-As_2_Ph_2_)],[25] which suggests a significant uranium to diarsene back-bonding-type interaction in **3** (see below), which would also be consistent with a diuranium(IV) formulation. Further support for the formulation of **3** comes from the crystal structure of **4**,[18] which crystallizes in a different crystal habit to **3**. In **4** the As=As distance of 2.2568(14) Å is shorter than the analogous distance in **3** and is characteristic of As_2_.[23a] Also, the U–As distances are shorter than in **3** at 3.0357(7) and 3.0497(8) Å that is consistent with the high charge load of As_2_.

**Figure 1:**
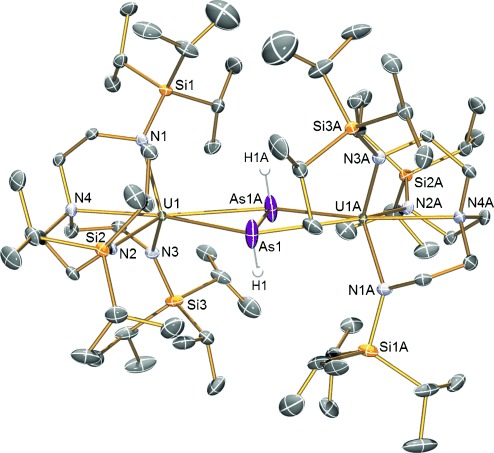
Molecular structure of 3 at 120 K with ellipsoids set at 50 % probability.[31] Non-arsenic-bound hydrogen atoms are omitted for clarity. An arbitrary pair of HAsAsH hydride positions corresponding to an *E* isomer have been selected, with the other pair omitted for clarity. Selected distances [Å]: U1–As1 3.1203(7), U1–As1A 3.1273(7), U1–N1 2.256(4), U1–N2 2.273(4), U1–N3 2.261(4), U1–N4 2.709(4), As1–As1A 2.4102(13).

The assignment of uranium(IV) ions in **3**, suggested by the solid-state metrical data, is also supported by magnetic measurements (Figure 2). A powdered sample of **3** exhibits a magnetic moment of 4.0 μ_B_ at 298 K, which decreases monotonously to a moment of 1.2 μ_B_ at 2 K and tends to zero as would be expected for uranium(IV), which at low temperature is a magnetic singlet with residual temperature-independent paramagnetism. The magnetic moment per uranium ion in **3** at 298 K (2.7 μ_B_) is lower than the theoretical value of 3.58 μ_B_ for uranium(IV), but this is common for uranium(IV).[26]

**Figure 2:**
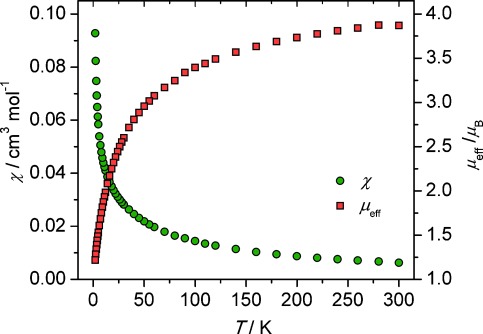
Temperature-dependent SQUID magnetization data for 3 plotted as *μ*_eff_ versus temperature (K) (□) and *χ* vs temperature (K) (○) over the range 1.8–298 K.

To probe the nature of the U–As interactions in **3**, we calculated the electronic structure of the full model using density functional theory (DFT).[18] With the *Z* isomer experimentally ruled out, our discussion focuses on the *E* isomer.[27] The geometry-optimized structure agrees well with experiment, with bond lengths and angles predicted to within 0.05 Å and 2°, respectively; the DFT model can thus be considered to present a qualitative description of the electronic structure of **3**. The calculated MDC_q_ charges and MDC_m_ spin densities at each uranium average +3.20 and −2.31, respectively, which suggests modest net donation of electron density to uranium(IV) from the ligands.[28] The arsenic MDC_q_ charges average −1.12, which is consistent with the HAsAsH fragment carrying a formal −2 charge overall, which is a requirement of being bonded to two uranium(IV) [U(Tren^TIPS^)]^+^ cations for charge neutrality. The calculated As–As Mayer bond order is 0.97, consistent with the As–As single bond suggested by the X-ray diffraction data, whereas the U–As Mayer bond orders average 0.34 and suggest polarized interactions; for comparison, calculated As–H, U–N_amide_, and U–N_amine_ Mayer bond orders average 0.92, 0.82, and 0.22, respectively.

The top four most energetic electrons in **3** are of essentially pure, non-bonding 5f character and constitute the top four quasi-degenerate (0.05 eV spread) α-spin highest occupied molecular orbitals (HOMOs), which are each singularly occupied. HOMO−4 in the α- and β-spin manifolds comprise the principal U–As interactions, and represent formal back-bonding from uranium to the π*-orbital of HAsAsH (Figure 3). As nitrogen-based orbital coefficients intrude into HOMO−4 of **3**, natural bond orbital (NBO) analyses were performed to obtain a localized, clear description of the U–As interactions. NBO analyses reveal highly polarized U–As interactions that comprise an average of 91.3 % As and 8.7 % U character, and these interactions are also found at the second-order level of perturbation. The arsenic components are essentially pure 4p character, whereas the uranium contributions are 53.2 % 5f and 45.3 % 6d character with no meaningful 7s or 7p contributions.

**Figure 3:**
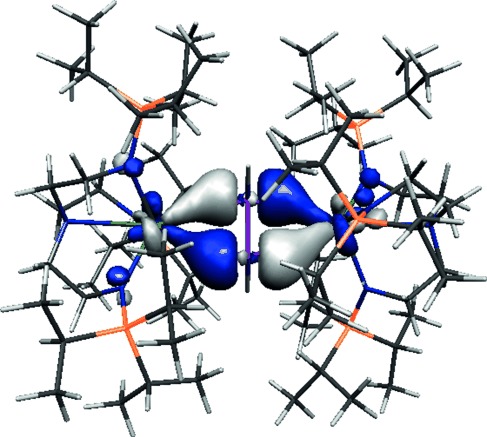
The α-spin Kohn–Sham HOMO−4 representation of the principal uranium–arsenic interaction in 3 at the 0.05 e Å^3^ level.

When considering the bonding of the HAsAsH fragment to two [U(Tren^TIPS^)] fragments, an a priori treatment yields two bonding extremes. On one hand, HAsAsH could donate electron density purely from its filled π-orbital to vacant orbitals on each uranium center, which would be assigned as formally trivalent, with no back-bonding and thus retain the As=As double bond. Alternatively, each uranium could formally engage in a back-bond-type interaction into the vacant π*-orbital of HAsAsH, leading to reduction to give two uranium(IV) centers and a HAsAsH dianion with an As–As single bond. Interestingly, all attempts to computationally model **3** as diuranium(III) with a formally neutral HAsAsH met with failure or converged instead to a diuranium(IV) HAsAsH-dianion spin-state formulation. Previous calculations on HAsAsH have predicted it to be an excellent π-acceptor ligand,[19, 29] and the combined characterization data for **3** clearly support the latter bonding picture, that is, in **3** HAsAsH can be considered as a diarsane-1,2-diide resulting from extensive electron transfer from uranium.

In summary, by careful control of reaction conditions we have been able to isolate the highly reactive HAsAsH unit between two sterically demanding uranium fragments, thus confirming the synthesis of a molecule first proposed over two centuries ago. The characterization data for **3** uniformly point to the HAsAsH unit being formally reduced to its dianionc form by the two uranium centers, in-line with the predicted excellent acceptor properties of HAsAsH. This study highlights the capacity of an f-block element, uranium, to bond in a manner that is reminiscent of d-block metals, though at one bonding extreme with highly polarized U–As bonding interactions. Complex **3** is an isoelectronic model for a π-alkene complex of uranium, which is a class of complex yet to be realized under any experimental conditions.[30]

## Supplementary material

As a service to our authors and readers, this journal provides supporting information supplied by the authors. Such materials are peer reviewed and may be re-organized for online delivery, but are not copy-edited or typeset. Technical support issues arising from supporting information (other than missing files) should be addressed to the authors.
