# Supplementary material for: Isolation of Elusive HAsAsH in a Crystalline Diuranium(IV) Complex
Source: Angew Chem Int Ed Engl. 2015 Oct 28;54(50):15250–4. doi: 10.1002/anie.201508600 (PMC4691330; doi:10.1002/anie.201508600)
Supplement: Supplementary file 1 [file anie0054-15250-SD1.pdf]

Angewandte Chemie
Eine Zeitschrift der Gesellschaft Deutscher Chemiker

Supporting Information

Isolation of Elusive HAsAsH in a Crystalline Diuranium(IV) Complex

Benedict M. Gardner, Gábor Balázs, Manfred Scheer, Ashley J. Wooles, Floriana Tuna, Eric J. L. McInnes, Jonathan McMaster, William Lewis, Alexander J. Blake, and Stephen T. Liddle**

anie_201508600_sm_miscellaneous_information.pdf

Experimental

General

All manipulations were carried out using Schlenk techniques, or an MBraun UniLab glovebox, under an atmosphere of dry nitrogen. Solvents were dried by passage through activated alumina towers and degassed before use. All solvents were stored over potassium mirrors except for THF which was stored over activated 4 Å sieves. Deuterated solvent was distilled from potassium, degassed by three freeze-pump-thaw cycles and stored under nitrogen. $[U(Tren^{TIPS})(THF)][BPh_4]$ (**1**), $[U(Tren^{TIPS})(AsH_2)]$ (**2**), and $KAsH_2$ were prepared according to literature methods.[1-3] ^1H and ^{29}Si NMR spectra were recorded on a Bruker 400 spectrometer operating at 400.2 and 79.5 MHz respectively; chemical shifts are quoted in ppm and are relative to TMS. FTIR spectra were recorded on a Bruker Alpha-Platinum ATR spectrometer. Variable-temperature magnetic moment data were recorded in an applied dc field of 0.1 T on a Quantum Design MPMS XL5 superconducting quantum interference device (SQUID) magnetometer. Samples were carefully checked for purity and data reproducibility. Care was taken to ensure complete thermalisation of the sample before each data point was measured and samples were immobilised in an eicosane matrix to prevent sample reorientation during measurements. Diamagnetic corrections were applied using tabulated Pascal constants and measurements were corrected for the effect of the blank sample holders (flame sealed Wilmad NMR tube and straw) and eicosane matrix. Solution magnetic moments were recorded at room temperature using the Evans method. Elemental microanalyses were carried out by Tong Liu at the University of Nottingham.

In the X-ray crystal structure of **3**, the arsenic-bound hydrogen atoms were initially located in the Fourier transform difference maps and refined with geometric restraints based on literature data and metrical data computed from the DFT geometry optimisations.

Preparation of [{U(Tren^{TIPS})}$_2$(μ-η^2:η^2-As$_2$H$_2$)] (3)

Method A: THF (20 ml) was added to a cold (−78 °C) mixture of **1** (0.62 g, 0.50 mmol) and finely ground $KAsH_2$ (81 mg, 0.70 mmol). The dark brown slurry was allowed to warm to room temperature whilst stirring (30 mins) to afford a dark brown suspension, which was then stirred at room temperature overnight.

Method B: Compound **2** (1.16 g, 1.25 mmol) was treated with $KAsH_2$ (81 mg, 0.70 mmol) in THF (10 ml) at −78 °C. The dark brown slurry was allowed to warm to room temperature whilst stirring (30 mins) to afford a dark brown suspension, which was then stirred at room temperature overnight.

Work-up: Solvent was removed *in vacuo* and the resulting pale brown solid was extracted into toluene (10 ml) and filtered to afford a dark brown solution. The volume of the solution was reduced *in vacuo* to 2 ml and stored at −30 °C for 24 hrs to afford dark brown crystals, which were isolated by filtration, washed with hexanes (3 × 2 ml) and dried *in vacuo* for 30 mins. Yield: 38 mg (8%). Assaying the toluene extract before crystallisation by ^{1}H NMR spectroscopy, with 2,4,6-$Bu^t_3C_6H_3$ as an internal standard, showed that **3** is obtained in 50% crude yield. The preparation of the D-analogue of **3**, **3D**, was accomplished by identical procedures with essentially identical outcomes except $KAsD_2$ (see below) was used in place of $KAsH_2$.

Characterisation data for ***3****:* Anal. calc'd for $C_{66}H_{152}As_2N_8Si_6U_2\cdot0.8C_7H_8$: C 44.65%; H 8.29%; N 5.82%. Found: C 45.03%; H 8.47%; N 6.24%. ^{1}H NMR (THF-d_8, 298 K): δ 5.35 (s, br, FWHM = 99 Hz, 126 H, Pr^i_3Si), 6.20 (s, br, FWHM = 66 Hz, 24 H, CH_2) ppm. As*H* resonance(s) not observed. ^{29}Si{^{1}H} NMR signal not observed due to lack of solubility. μ_{eff} (Evans method, THF-d_8, 298 K): 4.63 μ_B. FTIR (ATR-IR): ν 2069 (w), 2029 (w, br, As–H stretch), 1632 (w), 1581 (w), 1557 (w), 1540 (w), 1402 (s), 1302 (w), 1201 (w), 1046 (s), 931 (m), 904 (w), 672 (s), 652 (m), 633 (m), 575 (w), 561 (w), 541 (w), 518 (w) cm^{-1}.

Synthesis of KAsD₂

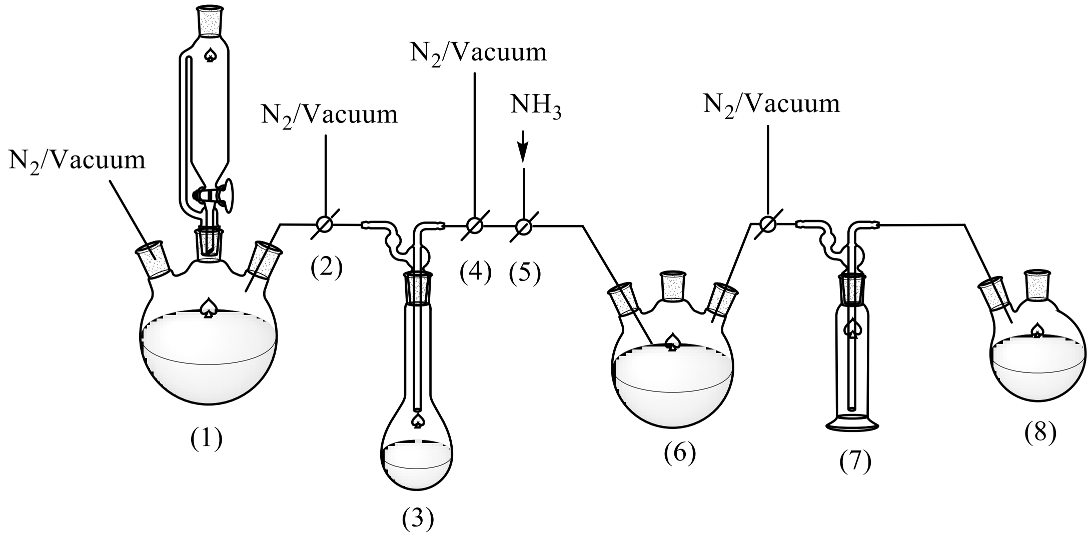

Figure S1. Apparatus used for the synthesis of $KAsD_2$.

$LiAlD_4$ (4.48 g, 106.71 mmol) was added in flask (1), the flask was cooled to −60 °C and THF (50 ml) was added. A solution of $AsCl_3$ (6.45 g, 3 ml, 35.57 mmol) in THF (5 ml) was filled into the dropping funnel. The cooling trap (3) was cooled with liquid nitrogen and the pressure in (1) and (3) was reduced to 550 mbar. The $AsCl_3$ solution was slowly added (dropwise) to the vigorously stirred $LiAlD_4$ solution. After the addition the temperature was slowly raised to −40 °C and the pressure slowly decreased to 0.1 mbar. After the evolution of AsD_3 was finished, potassium (0.9 g, 23.01 mmol) was placed in flask (6) and NH_3 (ca 150 ml) was condensed in the flask. In flask (8) a $KMnO_4$ solution in deoxygenated water was introduced. Subsequently, the three-way stopcock (4) was opened to the cooling trap containing AsD_3 and a slow stream of N_2 was passed through the apparatus starting from the three-way stopcock (2). The temperature of the cooling trap (3) was allowed to rise to room temperature, while the AsD_3 was passed through the blue K/NH_3 solution. The reaction is finished when the intense blue colour changes to bright yellow. The stream of N_2 is maintained for an additional 5 minutes. It has to be ensured that the cooling trap (3) reaches room temperature in order to ensure that all AsD_3 is transferred to (6). Subsequently, from flask (6) the NH_3 was slowly evaporated leading to a yellowish-light orange powder of $KAsD_2$ (2.30 g, 19.49 mmol). The reaction is quantitative with respect to K used.

Spectroscopy, Magnetism, and Crystallography

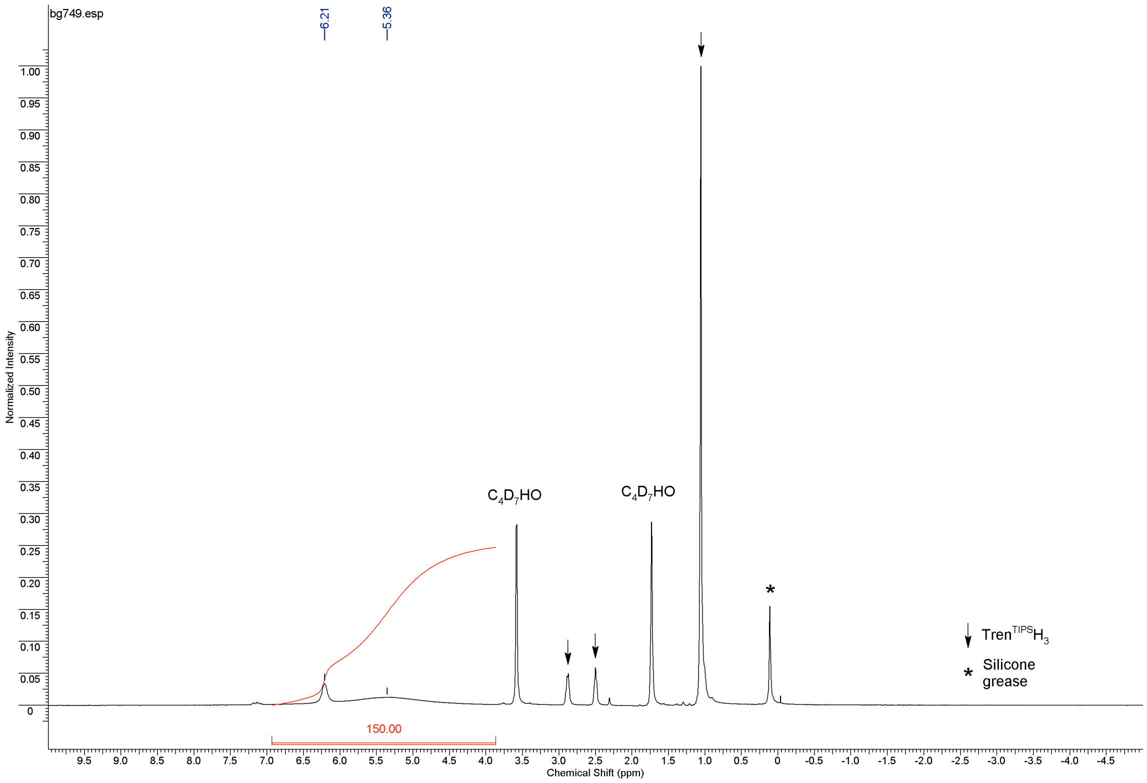

Figure S2. ***^{1}H NMR spectrum of 3 in D_8-THF.***

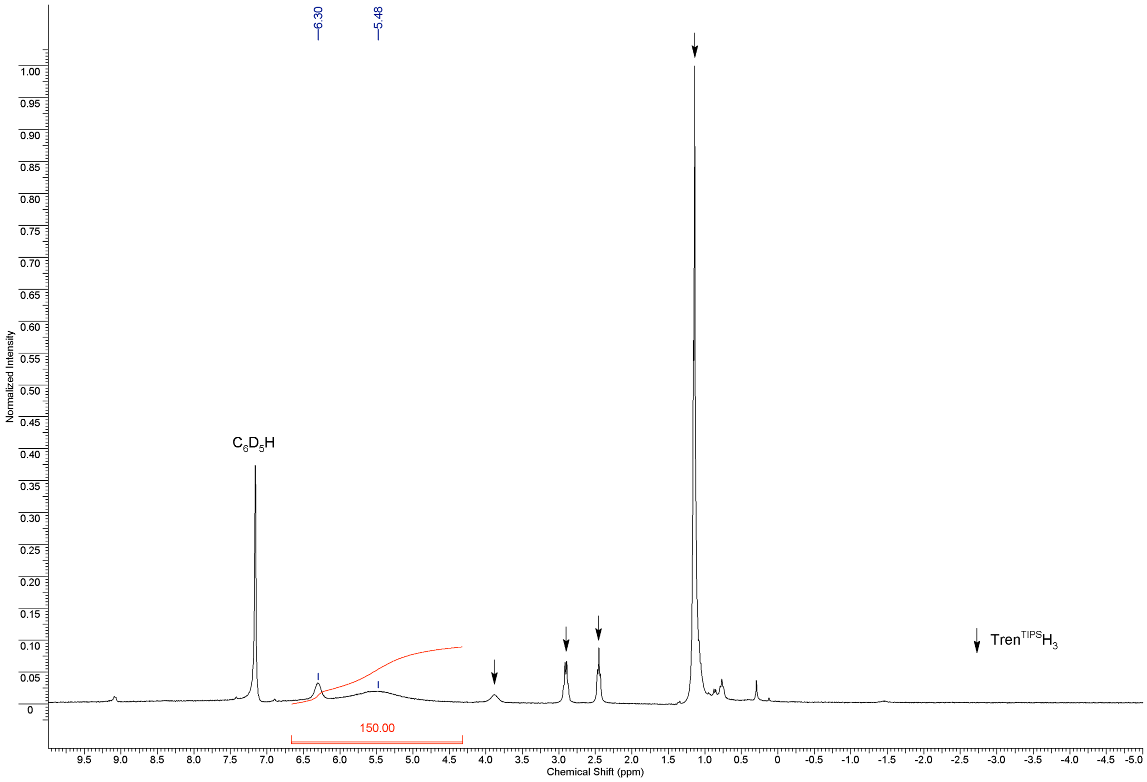

Figure S3. ***^{1}H NMR spectrum of 3D in D_6-benzene.***

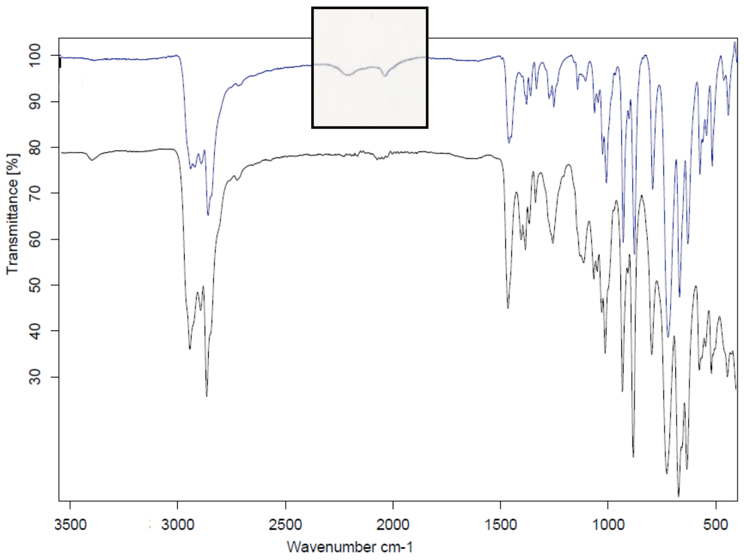

Figure S4. ATR-IR specta of 3 (blue) and 3D (black) with zoom in for As-H region of 3.

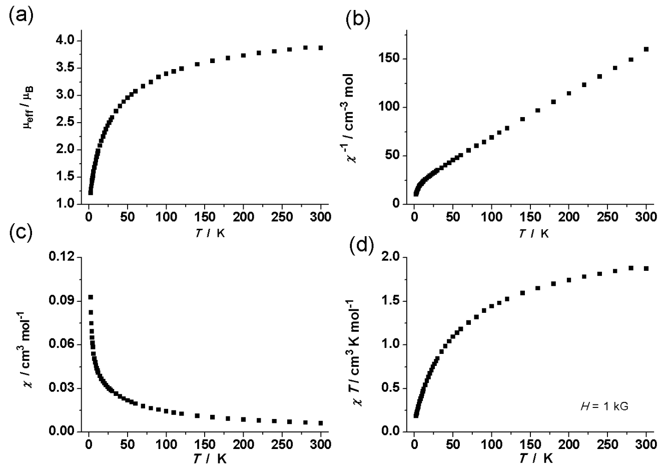

Figure S5. (a) μ_{eff} vs T, (b) 1/χ vs T, (c) χ vs T, and (d) χT vs T for 3.

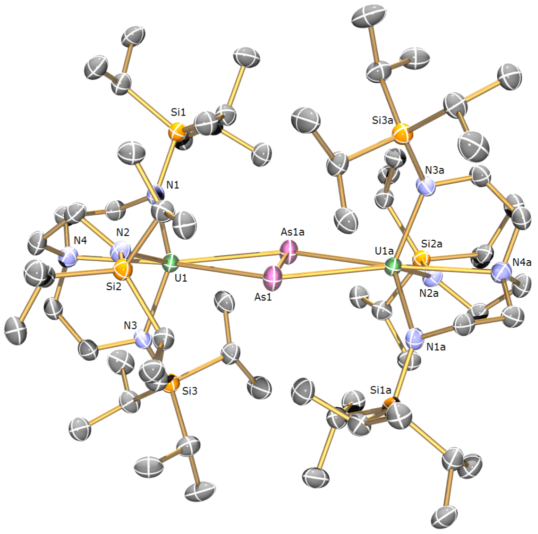

Figure S6. Molecular structure of 4.

Table S1. Experimental X-ray crystallographic details for 3 (CCDC-1403870).

Chemical formula	$C_{66}H_{152}As_2N_8Si_6U_2$
M_r	1852.39
Crystal system, space group	Monoclinic, *C*2/*c*
Temperature (K)	120
a, *b*, *c* (Å)	13.02406 (19), 23.0187 (3), 27.4687 (4)
β (°)	91.9142 (14)
V (Å^3)	8230.4 (2)
Z	4
Radiation type	Cu $K\alpha$
μ (mm^{-1})	13.02
Crystal size (mm)	0.20 × 0.14 × 0.07
Diffractometer	GV1000, Atlas diffractometer
Absorption correction	Multi-scan *CrysAlis PRO*, Agilent Technologies, Version 1.171.37.31 (release 14-01-2014 CrysAlis171 .NET) (compiled Jan 14 2014,18:38:05) Empirical absorption correction using spherical harmonics, implemented in SCALE3 ABSPACK scaling algorithm. Empirical absorption correction using spherical harmonics, implemented in SCALE3 ABSPACK scaling algorithm.
T_{min}, T_{max}	0.381, 1.000
No. of measured, independent and observed [$I > 2\sigma(I)$] reflections	31532, 8238, 7831
R_{int}	0.031
$(\sin \theta/\lambda)_{max}$ (Å^{-1})	0.624
$R[F^2 > 2\sigma(F^2)]$, $wR(F^2)$, S	0.035, 0.086, 1.04
No. of reflections	8238
No. of parameters	444
No. of restraints	734
H-atom treatment	H atoms treated by a mixture of independent and constrained refinement
$\Delta\rangle_{max}$, $\Delta\rangle_{min}$ (e Å^{-3})	4.50, -2.71

Computer programs: *CrysAlis PRO*, Agilent Technologies, Version 1.171.37.31 (release 14-01-2014 CrysAlis171 .NET) (compiled Jan 14 2014,18:38:05), *SHELXS* (Sheldrick, 2008), *SHELXL* (Sheldrick, 2008), Olex2 (Dolomanov *et al.*, 2009).

Table S2. Bond lengths (Å) and angles (°) for 3. Symmetry code: -x, y, -z+1/2.

U1—As1^{i}	3.1203 (7)	C18—H18	1.0000
U1—As1	3.1273 (7)	C18—C19	1.522 (9)
U1—N1	2.256 (4)	C18—C20	1.533 (9)
U1—N2	2.273 (4)	C19—H19A	0.9800
U1—N3	2.261 (4)	C19—H19B	0.9800
U1—N4	2.709 (4)	C19—H19C	0.9800
As1—U1^{i}	3.1203 (7)	C20—H20A	0.9800
As1—As1^{i}	2.4102 (13)	C20—H20B	0.9800
As1—H1A	1.32 (3)	C20—H20C	0.9800
As1—H1B	1.32 (3)	C21—H21A	0.9900
N1—Si1	1.752 (4)	C21—H21B	0.9900
N1—C10	1.490 (6)	C21—C22	1.510 (7)
Si1—C1	1.895 (6)	C22—H22A	0.9900
Si1—C4	1.910 (5)	C22—H22B	0.9900
Si1—C7	1.898 (6)	C22—N4	1.476 (7)
C1—H1	1.0000	N3—Si3	1.741 (4)
C1—C2	1.532 (8)	N3—C32	1.489 (7)
C1—C3	1.544 (8)	Si3—C23	1.896 (6)
C2—H2A	0.9800	Si3—C26	1.907 (7)
C2—H2B	0.9800	Si3—C29	1.949 (10)
C2—H2C	0.9800	Si3—C29A	1.892 (10)
C3—H3A	0.9800	C23—H23	1.0000
C3—H3B	0.9800	C23—C24	1.542 (9)
C3—H3C	0.9800	C23—C25	1.530 (8)
C4—H4	1.0000	C24—H24A	0.9800
C4—C5	1.547 (8)	C24—H24B	0.9800
C4—C6	1.532 (8)	C24—H24C	0.9800
C5—H5A	0.9800	C25—H25A	0.9800
C5—H5B	0.9800	C25—H25B	0.9800
C5—H5C	0.9800	C25—H25C	0.9800
C6—H6A	0.9800	C26—H26	1.0000
C6—H6B	0.9800	C26—C27	1.534 (9)
C6—H6C	0.9800	C26—C28	1.532 (10)
C7—H7A	1.0000	C27—H27A	0.9800
C7—H7B	1.0000	C27—H27B	0.9800
C7—C8	1.519 (8)	C27—H27C	0.9800
C7—C9	1.437 (11)	C28—H28A	0.9800
C7—C9A	1.533 (10)	C28—H28B	0.9800
C8—H8A	0.9800	C28—H28C	0.9800

C8—H8B	0.9800	C29—H29	1.0000
C8—H8C	0.9800	C29—C30	1.536 (12)
C10—H10A	0.9900	C29—C31	1.548 (12)
C10—H10B	0.9900	C29A—H29A	1.0000
C10—C11	1.517 (7)	C29A—C30A	1.525 (12)
C11—H11A	0.9900	C29A—C31A	1.538 (13)
C11—H11B	0.9900	C30—H30A	0.9800
C11—N4	1.484 (7)	C30—H30B	0.9800
N2—Si2	1.746 (4)	C30—H30C	0.9800
N2—C21	1.486 (6)	C30A—H30D	0.9800
Si2—C12	1.895 (5)	C30A—H30E	0.9800
Si2—C15	1.918 (6)	C30A—H30F	0.9800
Si2—C18	1.898 (6)	C31A—H31A	0.9800
C12—H12	1.0000	C31A—H31B	0.9800
C12—C13	1.540 (7)	C31A—H31C	0.9800
C12—C14	1.526 (8)	C32—H32A	0.9900
C13—H13A	0.9800	C32—H32B	0.9900
C13—H13B	0.9800	C32—C33	1.508 (7)
C13—H13C	0.9800	C33—H33A	0.9900
C14—H14A	0.9800	C33—H33B	0.9900
C14—H14B	0.9800	C33—N4	1.494 (7)
C14—H14C	0.9800	C9—H9A	0.9800
C15—H15	1.0000	C9—H9B	0.9800
C15—C16	1.539 (9)	C9—H9C	0.9800
C15—C17	1.537 (8)	C9A—H9AA	0.9800
C16—H16A	0.9800	C9A—H9AB	0.9800
C16—H16B	0.9800	C9A—H9AC	0.9800
C16—H16C	0.9800	C31—H31D	0.9800
C17—H17A	0.9800	C31—H31E	0.9800
C17—H17B	0.9800	C31—H31F	0.9800
C17—H17C	0.9800		
$As1^{i}$—U1—As1	45.38 (2)	Si2—C18—H18	106.8
N1—U1—As1	116.90 (11)	C19—C18—Si2	113.0 (4)
N1—U1—$As1^{i}$	99.66 (10)	C19—C18—H18	106.8
N1—U1—N2	109.51 (15)	C19—C18—C20	109.3 (6)
N1—U1—N3	105.95 (15)	C20—C18—Si2	113.8 (4)
N1—U1—N4	69.54 (14)	C20—C18—H18	106.8
N2—U1—As1	90.74 (11)	C18—C19—H19A	109.5
N2—U1—$As1^{i}$	135.45 (11)	C18—C19—H19B	109.5

N2—U1—N4	68.84 (14)	C18—C19—H19C	109.5
N3—U1—As1	122.81 (11)	H19A—C19—H19B	109.5
N3—U1—As1^{i}	93.44 (11)	H19A—C19—H19C	109.5
N3—U1—N2	109.29 (15)	H19B—C19—H19C	109.5
N3—U1—N4	69.68 (14)	C18—C20—H20A	109.5
N4—U1—As1	159.32 (9)	C18—C20—H20B	109.5
N4—U1—As1^{i}	155.29 (9)	C18—C20—H20C	109.5
U1^{i}—As1—U1	134.42 (2)	H20A—C20—H20B	109.5
U1^{i}—As1—H1A	80.6 (4)	H20A—C20—H20C	109.5
U1—As1—H1A	80.3 (4)	H20B—C20—H20C	109.5
U1—As1—H1B	84.1 (4)	N2—C21—H21A	109.9
U1^{i}—As1—H1B	84.4 (4)	N2—C21—H21B	109.9
As1^{i}—As1—U1^{i}	67.46 (3)	N2—C21—C22	109.1 (4)
As1^{i}—As1—U1	67.16 (3)	H21A—C21—H21B	108.3
As1^{i}—As1—H1A	69.8 (4)	C22—C21—H21A	109.9
As1^{i}—As1—H1B	69.9 (4)	C22—C21—H21B	109.9
H1A—As1—H1B	139.7 (8)	C21—C22—H22A	110.1
Si1—N1—U1	139.5 (2)	C21—C22—H22B	110.1
C10—N1—U1	106.4 (3)	H22A—C22—H22B	108.4
C10—N1—Si1	114.1 (3)	N4—C22—C21	107.9 (4)
N1—Si1—C1	111.2 (2)	N4—C22—H22A	110.1
N1—Si1—C4	109.8 (2)	N4—C22—H22B	110.1
N1—Si1—C7	108.6 (2)	Si3—N3—U1	135.6 (2)
C1—Si1—C4	111.1 (3)	C32—N3—U1	109.2 (3)
C1—Si1—C7	107.2 (3)	C32—N3—Si3	115.2 (3)
C7—Si1—C4	108.9 (3)	N3—Si3—C23	109.5 (2)
Si1—C1—H1	106.6	N3—Si3—C26	109.1 (3)
C2—C1—Si1	116.6 (4)	N3—Si3—C29	106.6 (4)
C2—C1—H1	106.6	N3—Si3—C29A	114.4 (4)
C2—C1—C3	108.3 (5)	C23—Si3—C26	111.7 (3)
C3—C1—Si1	111.6 (4)	C23—Si3—C29	100.9 (4)
C3—C1—H1	106.6	C26—Si3—C29	118.6 (4)
C1—C2—H2A	109.5	C29A—Si3—C23	114.7 (4)
C1—C2—H2B	109.5	C29A—Si3—C26	96.8 (4)
C1—C2—H2C	109.5	Si3—C23—H23	106.7
H2A—C2—H2B	109.5	C24—C23—Si3	111.8 (4)
H2A—C2—H2C	109.5	C24—C23—H23	106.7
H2B—C2—H2C	109.5	C25—C23—Si3	115.3 (4)
C1—C3—H3A	109.5	C25—C23—H23	106.7
C1—C3—H3B	109.5	C25—C23—C24	109.2 (5)

C1—C3—H3C	109.5	C23—C24—H24A	109.5
H3A—C3—H3B	109.5	C23—C24—H24B	109.5
H3A—C3—H3C	109.5	C23—C24—H24C	109.5
H3B—C3—H3C	109.5	H24A—C24—H24B	109.5
Si1—C4—H4	106.0	H24A—C24—H24C	109.5
C5—C4—Si1	113.9 (4)	H24B—C24—H24C	109.5
C5—C4—H4	106.0	C23—C25—H25A	109.5
C6—C4—Si1	115.1 (4)	C23—C25—H25B	109.5
C6—C4—H4	106.0	C23—C25—H25C	109.5
C6—C4—C5	109.0 (5)	H25A—C25—H25B	109.5
C4—C5—H5A	109.5	H25A—C25—H25C	109.5
C4—C5—H5B	109.5	H25B—C25—H25C	109.5
C4—C5—H5C	109.5	Si3—C26—H26	105.5
H5A—C5—H5B	109.5	C27—C26—Si3	116.2 (6)
H5A—C5—H5C	109.5	C27—C26—H26	105.5
H5B—C5—H5C	109.5	C28—C26—Si3	114.5 (4)
C4—C6—H6A	109.5	C28—C26—H26	105.5
C4—C6—H6B	109.5	C28—C26—C27	108.7 (6)
C4—C6—H6C	109.5	C26—C27—H27A	109.5
H6A—C6—H6B	109.5	C26—C27—H27B	109.5
H6A—C6—H6C	109.5	C26—C27—H27C	109.5
H6B—C6—H6C	109.5	H27A—C27—H27B	109.5
Si1—C7—H7A	105.6	H27A—C27—H27C	109.5
Si1—C7—H7B	100.3	H27B—C27—H27C	109.5
C8—C7—Si1	114.1 (4)	C26—C28—H28A	109.5
C8—C7—H7A	105.6	C26—C28—H28B	109.5
C8—C7—H7B	100.3	C26—C28—H28C	109.5
C8—C7—C9A	116.5 (7)	H28A—C28—H28B	109.5
C9—C7—Si1	117.5 (6)	H28A—C28—H28C	109.5
C9—C7—H7A	105.6	H28B—C28—H28C	109.5
C9—C7—C8	107.4 (7)	Si3—C29—H29	106.1
C9A—C7—Si1	119.9 (6)	C30—C29—Si3	110.7 (8)
C9A—C7—H7B	100.3	C30—C29—H29	106.1
C7—C8—H8A	109.5	C30—C29—C31	108.7 (9)
C7—C8—H8B	109.5	C31—C29—Si3	118.2 (7)
C7—C8—H8C	109.5	C31—C29—H29	106.1
H8A—C8—H8B	109.5	Si3—C29A—H29A	107.4
H8A—C8—H8C	109.5	C30A—C29A—Si3	116.1 (8)
H8B—C8—H8C	109.5	C30A—C29A—H29A	107.4
N1—C10—H10A	109.8	C30A—C29A—C31A	111.1 (10)

N1—C10—H10B	109.8	C31A—C29A—Si3	107.1 (7)
N1—C10—C11	109.4 (4)	C31A—C29A—H29A	107.4
H10A—C10—H10B	108.2	C29—C30—H30A	109.5
C11—C10—H10A	109.8	C29—C30—H30B	109.5
C11—C10—H10B	109.8	C29—C30—H30C	109.5
C10—C11—H11A	110.3	H30A—C30—H30B	109.5
C10—C11—H11B	110.3	H30A—C30—H30C	109.5
H11A—C11—H11B	108.5	H30B—C30—H30C	109.5
N4—C11—C10	107.3 (4)	C29A—C30A—H30D	109.5
N4—C11—H11A	110.3	C29A—C30A—H30E	109.5
N4—C11—H11B	110.3	C29A—C30A—H30F	109.5
Si2—N2—U1	138.7 (2)	H30D—C30A—H30E	109.5
C21—N2—U1	107.6 (3)	H30D—C30A—H30F	109.5
C21—N2—Si2	113.6 (3)	H30E—C30A—H30F	109.5
N2—Si2—C12	110.3 (2)	C29A—C31A—H31A	109.5
N2—Si2—C15	109.4 (2)	C29A—C31A—H31B	109.5
N2—Si2—C18	112.1 (2)	C29A—C31A—H31C	109.5
C12—Si2—C15	111.7 (2)	H31A—C31A—H31B	109.5
C12—Si2—C18	107.2 (3)	H31A—C31A—H31C	109.5
C18—Si2—C15	106.1 (3)	H31B—C31A—H31C	109.5
Si2—C12—H12	106.4	N3—C32—H32A	109.7
C13—C12—Si2	111.3 (4)	N3—C32—H32B	109.7
C13—C12—H12	106.4	N3—C32—C33	109.7 (4)
C14—C12—Si2	115.9 (4)	H32A—C32—H32B	108.2
C14—C12—H12	106.4	C33—C32—H32A	109.7
C14—C12—C13	109.8 (5)	C33—C32—H32B	109.7
C12—C13—H13A	109.5	C32—C33—H33A	109.9
C12—C13—H13B	109.5	C32—C33—H33B	109.9
C12—C13—H13C	109.5	H33A—C33—H33B	108.3
H13A—C13—H13B	109.5	N4—C33—C32	108.9 (4)
H13A—C13—H13C	109.5	N4—C33—H33A	109.9
H13B—C13—H13C	109.5	N4—C33—H33B	109.9
C12—C14—H14A	109.5	C11—N4—U1	106.4 (3)
C12—C14—H14B	109.5	C11—N4—C33	111.6 (4)
C12—C14—H14C	109.5	C22—N4—U1	107.1 (3)
H14A—C14—H14B	109.5	C22—N4—C11	112.9 (4)
H14A—C14—H14C	109.5	C22—N4—C33	111.9 (4)
H14B—C14—H14C	109.5	C33—N4—U1	106.3 (3)
Si2—C15—H15	105.7	C7—C9—H9A	109.5
C16—C15—Si2	114.5 (4)	C7—C9—H9B	109.5

C16—C15—H15	105.7	C7—C9—H9C	109.5
C17—C15—Si2	115.6 (4)	H9A—C9—H9B	109.5
C17—C15—H15	105.7	H9A—C9—H9C	109.5
C17—C15—C16	108.9 (5)	H9B—C9—H9C	109.5
C15—C16—H16A	109.5	C7—C9A—H9AA	109.5
C15—C16—H16B	109.5	C7—C9A—H9AB	109.5
C15—C16—H16C	109.5	C7—C9A—H9AC	109.5
H16A—C16—H16B	109.5	H9AA—C9A—H9AB	109.5
H16A—C16—H16C	109.5	H9AA—C9A—H9AC	109.5
H16B—C16—H16C	109.5	H9AB—C9A—H9AC	109.5
C15—C17—H17A	109.5	C29—C31—H31D	109.5
C15—C17—H17B	109.5	C29—C31—H31E	109.5
C15—C17—H17C	109.5	C29—C31—H31F	109.5
H17A—C17—H17B	109.5	H31D—C31—H31E	109.5
H17A—C17—H17C	109.5	H31D—C31—H31F	109.5
H17B—C17—H17C	109.5	H31E—C31—H31F	109.5

Table S3. Experimental X-ray crystallographic details for 4 (CCDC-1419459).

Chemical formula	$C_{66}H_{150}As_2N_8Si_6U_2$
M_r	1850.37
Crystal system, space group	Monoclinic, $P2_1/n$
Temperature (K)	120
a, *b*, *c* (Å)	12.9072 (5), 22.4152 (7), 15.0301 (6)
β (°)	113.904 (5)
V (Å^3)	3975.5 (3)
Z	2
Radiation type	Cu *K*a
μ (mm^{-1})	13.47
Crystal size (mm)	0.13 × 0.10 × 0.09
Diffractometer	GV1000, Atlas diffractometer
Absorption correction	Gaussian *CrysAlis PRO*, Agilent Technologies, Version 1.171.37.33 (release 27-03-2014 CrysAlis171 .NET) (compiled Mar 27 2014,17:12:48) Numerical absorption correction based on gaussian integration over a multifaceted crystal model Empirical absorption correction using spherical harmonics, implemented in SCALE3 ABSPACK scaling algorithm.
T_{min}, T_{max}	0.108, 0.252
No. of measured, independent and observed [$I > 2\sigma(I)$] reflections	17159, 7884, 6866
R_{int}	0.043
$(\sin \theta/\lambda)_{max}$ (Å^{-1})	0.624
$R[F^2 > 2\sigma(F^2)]$, $wR(F^2)$, S	0.046, 0.130, 1.05
No. of reflections	7884
No. of parameters	397
H-atom treatment	H-atom parameters constrained
$\Delta\rangle_{max}$, $\Delta\rangle_{min}$ (e Å^{-3})	3.76, -1.47

Computer programs: *CrysAlis PRO*, Agilent Technologies, Version 1.171.37.33 (release 27-03-2014 CrysAlis171 .NET) (compiled Mar 27 2014,17:12:48), *SHELXS* (Sheldrick, 2008), *SHELXL* (Sheldrick, 2008), Olex2 (Dolomanov *et al.*, 2009).

Table S4. Bond lengths (Å) and angles (°) for 4. Symmetry code: -x, -y, -z.

U1—As1	3.0357 (7)	C15—C16	1.524 (11)
U1—As1^{i}	3.0497 (8)	C15—C17	1.530 (11)
U1—N1	2.270 (5)	C16—H16A	0.9800
U1—N2	2.278 (5)	C16—H16B	0.9800
U1—N3	2.266 (5)	C16—H16C	0.9800
U1—N4	2.685 (5)	C17—H17A	0.9800
As1—U1^{i}	3.0497 (7)	C17—H17B	0.9800
As1—As1^{i}	2.2568 (14)	C17—H17C	0.9800
N1—Si1	1.744 (5)	C18—H18	1.0000
N1—C10	1.490 (7)	C18—C19	1.532 (11)
Si1—C1	1.894 (7)	C18—C20	1.541 (11)
Si1—C4	1.917 (7)	C19—H19A	0.9800
Si1—C7	1.912 (7)	C19—H19B	0.9800
C1—H1	1.0000	C19—H19C	0.9800
C1—C2	1.545 (10)	C20—H20A	0.9800
C1—C3	1.558 (10)	C20—H20B	0.9800
C2—H2A	0.9800	C20—H20C	0.9800
C2—H2B	0.9800	C21—H21A	0.9900
C2—H2C	0.9800	C21—H21B	0.9900
C3—H3A	0.9800	C21—C22	1.520 (10)
C3—H3B	0.9800	C22—H22A	0.9900
C3—H3C	0.9800	C22—H22B	0.9900
C4—H4	1.0000	C22—N4	1.480 (9)
C4—C5	1.520 (11)	N3—Si3	1.753 (6)
C4—C6	1.523 (10)	N3—C32	1.499 (8)
C5—H5A	0.9800	Si3—C23	1.889 (7)
C5—H5B	0.9800	Si3—C26	1.923 (6)
C5—H5C	0.9800	Si3—C29	1.900 (7)
C6—H6A	0.9800	C23—H23	1.0000
C6—H6B	0.9800	C23—C24	1.527 (10)
C6—H6C	0.9800	C23—C25	1.541 (10)
C7—H7	1.0000	C24—H24A	0.9800
C7—C8	1.526 (10)	C24—H24B	0.9800
C7—C9	1.526 (10)	C24—H24C	0.9800
C8—H8A	0.9800	C25—H25A	0.9800
C8—H8B	0.9800	C25—H25B	0.9800
C8—H8C	0.9800	C25—H25C	0.9800
C9—H9A	0.9800	C26—H26	1.0000
C9—H9B	0.9800	C26—C27	1.519 (10)

C9—H9C	0.9800	C26—C28	1.540 (10)
C10—H10A	0.9900	C27—H27A	0.9800
C10—H10B	0.9900	C27—H27B	0.9800
C10—C11	1.477 (10)	C27—H27C	0.9800
C11—H11A	0.9900	C28—H28A	0.9800
C11—H11B	0.9900	C28—H28B	0.9800
C11—N4	1.469 (8)	C28—H28C	0.9800
N2—Si2	1.741 (5)	C29—H29	1.0000
N2—C21	1.471 (8)	C29—C30	1.553 (9)
Si2—C12	1.889 (7)	C29—C31	1.515 (10)
Si2—C15	1.909 (7)	C30—H30A	0.9800
Si2—C18	1.906 (7)	C30—H30B	0.9800
C12—H12	1.0000	C30—H30C	0.9800
C12—C13	1.530 (10)	C31—H31A	0.9800
C12—C14	1.550 (10)	C31—H31B	0.9800
C13—H13A	0.9800	C31—H31C	0.9800
C13—H13B	0.9800	C32—H32A	0.9900
C13—H13C	0.9800	C32—H32B	0.9900
C14—H14A	0.9800	C32—C33	1.506 (10)
C14—H14B	0.9800	C33—H33A	0.9900
C14—H14C	0.9800	C33—H33B	0.9900
C15—H15	1.0000	C33—N4	1.486 (9)
As1—U1—As1^{i}	43.54 (3)	C17—C15—H15	104.2
N1—U1—As1^{i}	90.70 (13)	C15—C16—H16A	109.5
N1—U1—As1	125.05 (13)	C15—C16—H16B	109.5
N1—U1—N2	110.74 (19)	C15—C16—H16C	109.5
N1—U1—N4	69.58 (18)	H16A—C16—H16B	109.5
N2—U1—As1^{i}	109.31 (13)	H16A—C16—H16C	109.5
N2—U1—As1	112.37 (13)	H16B—C16—H16C	109.5
N2—U1—N4	69.53 (18)	C15—C17—H17A	109.5
N3—U1—As1^{i}	129.20 (14)	C15—C17—H17B	109.5
N3—U1—As1	89.65 (14)	C15—C17—H17C	109.5
N3—U1—N1	109.09 (19)	H17A—C17—H17B	109.5
N3—U1—N2	106.34 (19)	H17A—C17—H17C	109.5
N3—U1—N4	70.31 (19)	H17B—C17—H17C	109.5
N4—U1—As1^{i}	157.21 (12)	Si2—C18—H18	106.0
N4—U1—As1	159.06 (12)	C19—C18—Si2	115.4 (5)
U1—As1—U1^{i}	136.46 (3)	C19—C18—H18	106.0
As1^{i}—As1—U1	68.56 (3)	C19—C18—C20	106.9 (6)

As1^{i}—As1—U1^{i}	67.90 (3)	C20—C18—Si2	115.9 (5)
Si1—N1—U1	137.0 (3)	C20—C18—H18	106.0
C10—N1—U1	109.0 (4)	C18—C19—H19A	109.5
C10—N1—Si1	113.9 (4)	C18—C19—H19B	109.5
N1—Si1—C1	107.7 (3)	C18—C19—H19C	109.5
N1—Si1—C4	111.2 (3)	H19A—C19—H19B	109.5
N1—Si1—C7	112.7 (3)	H19A—C19—H19C	109.5
C1—Si1—C4	112.9 (3)	H19B—C19—H19C	109.5
C1—Si1—C7	107.1 (3)	C18—C20—H20A	109.5
C7—Si1—C4	105.2 (3)	C18—C20—H20B	109.5
Si1—C1—H1	106.8	C18—C20—H20C	109.5
C2—C1—Si1	115.4 (5)	H20A—C20—H20B	109.5
C2—C1—H1	106.8	H20A—C20—H20C	109.5
C2—C1—C3	108.9 (6)	H20B—C20—H20C	109.5
C3—C1—Si1	111.7 (5)	N2—C21—H21A	109.7
C3—C1—H1	106.8	N2—C21—H21B	109.7
C1—C2—H2A	109.5	N2—C21—C22	109.6 (5)
C1—C2—H2B	109.5	H21A—C21—H21B	108.2
C1—C2—H2C	109.5	C22—C21—H21A	109.7
H2A—C2—H2B	109.5	C22—C21—H21B	109.7
H2A—C2—H2C	109.5	C21—C22—H22A	110.3
H2B—C2—H2C	109.5	C21—C22—H22B	110.3
C1—C3—H3A	109.5	H22A—C22—H22B	108.5
C1—C3—H3B	109.5	N4—C22—C21	107.3 (5)
C1—C3—H3C	109.5	N4—C22—H22A	110.3
H3A—C3—H3B	109.5	N4—C22—H22B	110.3
H3A—C3—H3C	109.5	Si3—N3—U1	138.5 (3)
H3B—C3—H3C	109.5	C32—N3—U1	105.9 (4)
Si1—C4—H4	105.8	C32—N3—Si3	115.3 (4)
C5—C4—Si1	114.3 (5)	N3—Si3—C23	108.3 (3)
C5—C4—H4	105.8	N3—Si3—C26	108.8 (3)
C5—C4—C6	109.9 (6)	N3—Si3—C29	111.0 (3)
C6—C4—Si1	114.4 (5)	C23—Si3—C26	110.7 (3)
C6—C4—H4	105.8	C23—Si3—C29	107.2 (3)
C4—C5—H5A	109.5	C29—Si3—C26	110.8 (3)
C4—C5—H5B	109.5	Si3—C23—H23	105.4
C4—C5—H5C	109.5	C24—C23—Si3	112.7 (5)
H5A—C5—H5B	109.5	C24—C23—H23	105.4
H5A—C5—H5C	109.5	C24—C23—C25	110.7 (6)
H5B—C5—H5C	109.5	C25—C23—Si3	116.3 (5)

C4—C6—H6A	109.5	C25—C23—H23	105.4
C4—C6—H6B	109.5	C23—C24—H24A	109.5
C4—C6—H6C	109.5	C23—C24—H24B	109.5
H6A—C6—H6B	109.5	C23—C24—H24C	109.5
H6A—C6—H6C	109.5	H24A—C24—H24B	109.5
H6B—C6—H6C	109.5	H24A—C24—H24C	109.5
Si1—C7—H7	106.8	H24B—C24—H24C	109.5
C8—C7—Si1	114.9 (5)	C23—C25—H25A	109.5
C8—C7—H7	106.8	C23—C25—H25B	109.5
C9—C7—Si1	111.3 (5)	C23—C25—H25C	109.5
C9—C7—H7	106.8	H25A—C25—H25B	109.5
C9—C7—C8	109.7 (6)	H25A—C25—H25C	109.5
C7—C8—H8A	109.5	H25B—C25—H25C	109.5
C7—C8—H8B	109.5	Si3—C26—H26	106.2
C7—C8—H8C	109.5	C27—C26—Si3	115.2 (5)
H8A—C8—H8B	109.5	C27—C26—H26	106.2
H8A—C8—H8C	109.5	C27—C26—C28	110.1 (6)
H8B—C8—H8C	109.5	C28—C26—Si3	112.3 (5)
C7—C9—H9A	109.5	C28—C26—H26	106.2
C7—C9—H9B	109.5	C26—C27—H27A	109.5
C7—C9—H9C	109.5	C26—C27—H27B	109.5
H9A—C9—H9B	109.5	C26—C27—H27C	109.5
H9A—C9—H9C	109.5	H27A—C27—H27B	109.5
H9B—C9—H9C	109.5	H27A—C27—H27C	109.5
N1—C10—H10A	109.9	H27B—C27—H27C	109.5
N1—C10—H10B	109.9	C26—C28—H28A	109.5
H10A—C10—H10B	108.3	C26—C28—H28B	109.5
C11—C10—N1	109.0 (5)	C26—C28—H28C	109.5
C11—C10—H10A	109.9	H28A—C28—H28B	109.5
C11—C10—H10B	109.9	H28A—C28—H28C	109.5
C10—C11—H11A	109.5	H28B—C28—H28C	109.5
C10—C11—H11B	109.5	Si3—C29—H29	106.2
H11A—C11—H11B	108.1	C30—C29—Si3	113.7 (4)
N4—C11—C10	110.7 (5)	C30—C29—H29	106.2
N4—C11—H11A	109.5	C31—C29—Si3	115.3 (5)
N4—C11—H11B	109.5	C31—C29—H29	106.2
Si2—N2—U1	135.8 (3)	C31—C29—C30	108.5 (6)
C21—N2—U1	109.3 (4)	C29—C30—H30A	109.5
C21—N2—Si2	114.9 (4)	C29—C30—H30B	109.5
N2—Si2—C12	106.3 (3)	C29—C30—H30C	109.5

N2—Si2—C15	117.0 (3)	H30A—C30—H30B	109.5
N2—Si2—C18	110.2 (3)	H30A—C30—H30C	109.5
C12—Si2—C15	109.2 (3)	H30B—C30—H30C	109.5
C12—Si2—C18	107.0 (3)	C29—C31—H31A	109.5
C18—Si2—C15	106.7 (3)	C29—C31—H31B	109.5
Si2—C12—H12	106.1	C29—C31—H31C	109.5
C13—C12—Si2	115.6 (5)	H31A—C31—H31B	109.5
C13—C12—H12	106.1	H31A—C31—H31C	109.5
C13—C12—C14	108.6 (7)	H31B—C31—H31C	109.5
C14—C12—Si2	113.7 (5)	N3—C32—H32A	109.8
C14—C12—H12	106.1	N3—C32—H32B	109.8
C12—C13—H13A	109.5	N3—C32—C33	109.3 (5)
C12—C13—H13B	109.5	H32A—C32—H32B	108.3
C12—C13—H13C	109.5	C33—C32—H32A	109.8
H13A—C13—H13B	109.5	C33—C32—H32B	109.8
H13A—C13—H13C	109.5	C32—C33—H33A	110.2
H13B—C13—H13C	109.5	C32—C33—H33B	110.2
C12—C14—H14A	109.5	H33A—C33—H33B	108.5
C12—C14—H14B	109.5	N4—C33—C32	107.3 (5)
C12—C14—H14C	109.5	N4—C33—H33A	110.2
H14A—C14—H14B	109.5	N4—C33—H33B	110.2
H14A—C14—H14C	109.5	C11—N4—U1	106.1 (4)
H14B—C14—H14C	109.5	C11—N4—C22	111.8 (5)
Si2—C15—H15	104.2	C11—N4—C33	112.3 (6)
C16—C15—Si2	117.1 (6)	C22—N4—U1	107.5 (4)
C16—C15—H15	104.2	C22—N4—C33	112.3 (6)
C16—C15—C17	108.4 (6)	C33—N4—U1	106.3 (4)
C17—C15—Si2	116.9 (5)		

Density Functional Theory Calculations

General

Unrestricted geometry optimisations were performed for the full model of **3** using coordinates derived from the X-ray crystal structure; both *E* and *Z* isomers were considered, but since the *E* isomer was found to be the most stable subsequent investigations focussed on this isomer. No constraints were imposed on the structures during the geometry optimisations. The calculations were performed using the Amsterdam Density Functional (ADF) suite version 2012.01.[4,5] The DFT

geometry optimisations employed Slater type orbital (STO) triple-ζ-plus polarisation all-electron basis sets (from the ZORA/TZP database of the ADF suite). Scalar relativistic approaches were used within the ZORA Hamiltonian for the inclusion of relativistic effects and the local density approximation (LDA) with the correlation potential due to Vosko et al[6] was used in all of the calculations. Gradient corrections were performed using the functionals of Becke[7] and Perdew.[8] MOLEKEL[9] was used to prepare the three-dimensional plot of the electron density. Natural Bond Order (NBO) analyses were carried out with NBO 5.0.[10]

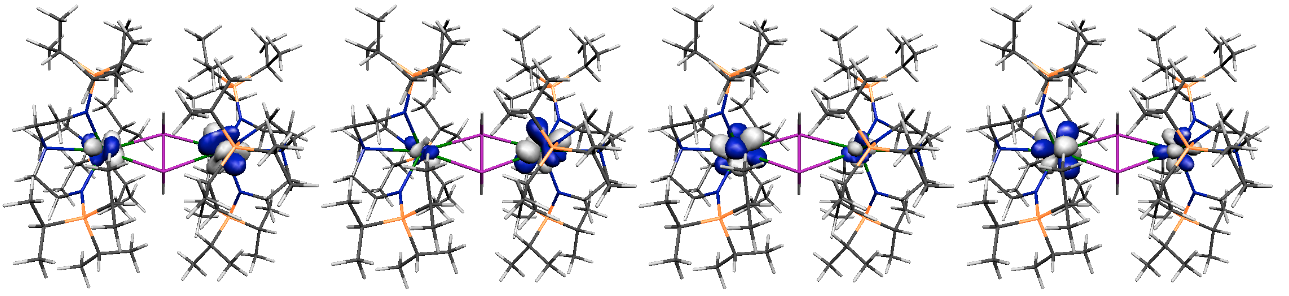

(a) (b) (c) (d)

Figure S7. The top four α-spin Kohn Sham molecular orbitals of 3: (a) HOMO (471a, −2.944 eV), (b) HOMO−1 (470a, −2.951), (c) HOMO−2 (469a, −2.977 eV), (d) HOMO−3 (468a, −2.989).

Table S5. Final coordinates and single point energy of the E isomer of 3 after geometry optimisation.

1.C	4.208630	0.936163	-6.513490
2.C	1.081636	-1.122586	-5.819694
3.C	0.651898	1.365979	-5.701500
4.C	4.369861	0.202329	-5.163745
5.C	1.153532	0.131957	-4.924978
6.C	-3.386808	-0.599204	-4.847466
7.C	5.696831	0.629624	-4.498591
8.C	-3.692136	1.868348	-4.389044
9.C	3.087397	3.185818	-3.923419
10.C	-6.869168	-0.155308	-3.754097
11.C	-3.387302	0.493036	-3.759436
12.C	3.374102	-2.356061	-3.463361
13.C	1.910347	-6.296079	-3.228783
14.C	4.762785	-2.688120	-2.927595
15.C	2.814943	1.969700	-3.009945
16.C	-0.233070	-3.426956	-2.942480
17.C	-4.750590	-2.740744	-2.380918
18.C	-6.315055	0.360782	-2.407470
19.C	-1.658240	-5.327518	-2.076037
20.C	-1.065050	3.833289	-2.178073
21.C	0.639805	5.670354	-1.835186
22.C	1.675619	-6.552902	-1.726113
23.C	0.939819	-7.899539	-1.544063
24.C	3.745405	2.051823	-1.787698
25.C	-0.593854	-4.272615	-1.710094
26.C	-3.986916	-1.665981	-1.576095
27.C	-7.170414	-0.194989	-1.247544
28.C	-2.987714	6.620980	-0.990370
29.C	4.450044	-4.307499	-1.089229
30.C	-0.140613	4.522303	-1.161381
31.C	-4.471169	2.658544	-1.067209
32.C	5.787909	-2.269441	-0.708071
33.C	5.396192	-0.856905	-0.291794
34.C	-1.283128	8.007378	0.245211
35.C	-4.171912	-1.938646	-0.073987
36.C	-5.442781	2.899879	0.084665
37.C	3.404358	-4.370065	0.017393
38.C	-2.062241	6.673138	0.242869
39.C	0.188161	-5.773856	0.887005

40.C	5.729354	2.126457	1.511312	91.H	0.959049	-6.260301	-3.781556
41.C	0.476216	5.576844	1.681021	92.H	-3.532637	2.692734	-3.682482
42.C	-4.271645	4.274606	1.774531	93.H	2.511954	-7.105653	-3.676986
43.C	-0.669188	-4.775892	1.684648	94.H	5.855350	0.159756	-3.517120
44.C	1.245745	-6.411291	1.811805	95.H	0.238047	-4.027480	-3.732985
45.C	1.556147	4.488590	1.811177	96.H	5.197898	-3.579463	-3.418804
46.C	4.505731	1.542946	2.247551	97.H	2.942895	4.122631	-3.361355
47.C	-2.830777	4.229587	2.268676	98.H	2.434341	-5.348991	-3.423919
48.C	-5.345971	2.155252	2.446222	99.H	-2.355641	0.548577	-3.360297
49.C	-4.861325	0.710941	2.418601	100.H	-4.633179	-2.633526	-3.466602
50.C	6.089324	-1.184358	2.888790	101.H	2.700186	-3.197327	-3.225757
51.C	0.015848	6.029630	3.081410	102.H	-1.134217	-2.966068	-3.378086
52.C	5.984119	-2.704420	3.138038	103.H	5.428284	-1.839221	-3.125154
53.C	2.296516	-2.281126	2.963646	104.H	-1.275096	-6.054279	-2.808503
54.C	4.323399	2.240303	3.610099	105.H	-0.496018	3.512499	-3.065866
55.C	2.921107	-0.920923	3.319662	106.H	1.185881	5.300826	-2.717790
56.C	-4.391232	-2.522634	3.700102	107.H	-2.540830	-4.848890	-2.529158
57.C	-2.944669	-2.005756	3.841629	108.H	1.778913	2.062961	-2.631892
58.C	6.742356	-0.494153	4.106061	109.H	0.457914	-2.599850	-2.711817
59.C	-0.572008	1.795081	4.008438	110.H	-5.827933	-2.715016	-2.160922
60.C	-0.967706	0.354206	4.374355	111.H	-1.868961	4.495212	-2.529146
61.C	-2.160974	-2.889939	4.831926	112.H	-6.469153	1.456130	-2.418171
62.C	3.228304	-0.901342	4.833855	113.H	-0.029546	6.471197	-2.184395
63.C	-3.942715	0.523739	5.558489	114.H	1.477060	-8.705713	-2.073321
64.C	-3.684270	1.992542	5.957122	115.H	-0.081015	-7.869521	-1.955414
65.C	-0.496122	0.069418	5.817764	116.H	-4.394602	-3.746742	-2.106829
66.C	-3.951748	-0.368367	6.819072	117.H	4.803573	1.975913	-2.076068
67.H	5.103883	0.782109	-7.141577	118.H	-2.412374	6.697595	-1.925547
68.H	3.343050	0.574047	-7.085778	119.H	-4.999908	2.790046	-2.023759
69.H	0.081039	-1.219881	-6.273351	120.H	4.062344	-4.844002	-1.963962
70.H	1.806293	-1.077870	-6.648261	121.H	-1.538980	2.923017	-1.774630
71.H	4.090501	2.022229	-6.382507	122.H	-2.910317	-1.789597	-1.799521
72.H	1.327857	1.647151	-6.523435	123.H	-8.218299	0.139197	-1.338018
73.H	-0.333410	1.157063	-6.151958	124.H	6.718323	-2.280404	-1.307063
74.H	-2.725405	-0.307651	-5.680839	125.H	-2.004412	-5.892512	-1.198478
75.H	4.471936	-0.869672	-5.414268	126.H	2.672956	-6.684870	-1.264508
76.H	-3.033504	2.052560	-5.254302	127.H	-7.183787	-1.295063	-1.248960
77.H	1.273419	-2.044958	-5.256608	128.H	1.377845	6.126989	-1.159651
78.H	6.558043	0.364277	-5.135806	129.H	3.619944	3.019583	-1.274409
79.H	-4.388064	-0.763422	-5.274052	130.H	-3.701710	7.462454	-0.982156
80.H	0.529744	2.244100	-5.052367	131.H	-3.574293	5.692164	-1.046713
81.H	2.431918	3.224192	-4.802365	132.H	5.225210	-0.256819	-1.201201
82.H	-6.373289	0.311758	-4.616686	133.H	-3.711859	3.462127	-1.050521
83.H	-4.728510	1.933930	-4.755731	134.H	3.539108	1.275800	-1.036793
84.H	3.408333	-2.316429	-4.563582	135.H	-1.052406	-3.586057	-0.975029
85.H	4.126223	3.191279	-4.285513	136.H	5.402764	-4.787187	-0.795369
86.H	5.736226	1.718053	-4.343523	137.H	-0.992978	-1.104618	-1.003557
87.H	0.443250	-0.030042	-4.090563	138.H	0.598669	3.761063	-0.849866
88.H	-3.022226	-1.563884	-4.468250	139.H	-0.583630	8.076866	-0.601926
89.H	-7.947530	0.068972	-3.835602	140.H	0.861090	-8.197717	-0.489064
90.H	-6.758759	-1.245402	-3.856294	141.H	-6.807360	0.119981	-0.258417

142.H	-6.008963	3.842556	-0.039576
143.H	-1.979791	8.859314	0.159404
144.H	5.967278	-2.863274	0.197663
145.H	-6.166868	2.076869	0.112850
146.H	6.257066	-0.387968	0.211292
147.H	-5.225669	-1.846442	0.225693
148.H	-3.854180	-2.963945	0.175596
149.H	3.306665	-5.409718	0.367512
150.H	-0.481619	-6.589793	0.552322
151.H	5.793219	1.780158	0.471703
152.H	-3.579050	-1.264637	0.561100
153.H	-0.700786	8.153146	1.166100
154.H	3.777146	-3.806448	0.891177
155.H	-2.717639	6.700959	1.133897
156.H	-4.318963	4.936833	0.901184
157.H	2.009653	4.231891	0.843568
158.H	0.945617	6.452865	1.193152
159.H	1.874615	-7.152550	1.296141
160.H	-1.523540	-4.400515	1.103803
161.H	5.673972	3.227734	1.482605
162.H	1.148910	1.163682	1.398727
163.H	-5.160933	0.255821	1.459394
164.H	3.616496	1.793004	1.638162
165.H	6.675632	1.866236	2.011262
166.H	-0.080360	-3.901969	1.999178
167.H	6.797207	-1.049509	2.048606
168.H	1.911587	-5.647915	2.242208
169.H	1.908817	-2.345466	1.932328
170.H	-6.447002	2.226618	2.358796
171.H	1.140552	3.562559	2.234653
172.H	5.518805	-3.240224	2.297485
173.H	-4.964729	4.675591	2.538456
174.H	2.369332	4.819932	2.478666
175.H	0.761328	-6.927082	2.658463
176.H	-1.073148	-5.248149	2.596112
177.H	-2.551887	5.220971	2.659943
178.H	-0.747798	6.820980	3.044169
179.H	-2.460563	-2.125155	2.853094
180.H	-4.932344	-2.027152	2.883511
181.H	-2.773748	3.549644	3.135801
182.H	6.981745	-3.146937	3.301552
183.H	3.010396	-3.106804	3.093447
184.H	-5.399503	0.143615	3.194832
185.H	-0.760643	2.071063	2.956998
186.H	4.311761	3.335569	3.479476
187.H	2.153310	-0.145890	3.129076
188.H	-5.056602	2.601949	3.406026
189.H	-4.395454	-3.604062	3.482305
190.H	0.866081	6.423453	3.663835
191.H	-0.402345	5.187889	3.654199
192.H	1.432575	-2.492999	3.614493
193.H	6.942720	0.571331	3.923788
194.H	5.385015	-2.926258	4.034308
195.H	3.376745	1.963753	4.095109
196.H	5.139791	2.007498	4.310653
197.H	-0.406694	-0.325110	3.703403
198.H	7.708614	-0.972352	4.345035
199.H	0.507733	1.952585	4.163963
200.H	-2.258292	-3.952406	4.551701
201.H	-4.973414	-2.379775	4.624242
202.H	-1.099627	2.532984	4.629325
203.H	-1.087091	-2.656597	4.837810
204.H	6.116029	-0.565331	5.008089
205.H	3.946617	-1.688953	5.105882
206.H	3.643463	0.055398	5.176143
207.H	-3.664854	2.669724	5.090731
208.H	-4.970454	0.482082	5.150262
209.H	2.310574	-1.091170	5.413006
210.H	-2.531347	-2.793502	5.864118
211.H	0.589930	0.232734	5.906288
212.H	-0.703710	-0.958282	6.142039
213.H	-2.721364	2.104944	6.478299
214.H	-4.465700	2.357157	6.645749
215.H	-0.980748	0.746549	6.536924
216.H	-4.248377	-1.402924	6.596323
217.H	-2.966690	-0.403081	7.308613
218.H	-4.667900	0.024907	7.561815
219.As	0.658081	1.039938	-0.054137
220.As	-0.543569	-1.044748	0.471823
221.N	2.880842	-1.098356	-2.844052
222.N	4.666957	-2.886830	-1.460190
223.N	-3.831792	1.321180	-0.938059
224.N	2.121261	-3.802542	-0.474523
225.N	4.174159	-0.891402	0.554411
226.N	-1.940923	3.763392	1.171850
227.N	-4.677787	2.909186	1.357384
228.N	-3.385894	0.671091	2.581328
229.Si	2.831539	0.296374	-3.969481
230.Si	-4.397118	0.129580	-2.145627
231.Si	0.885055	-5.050735	-0.763766
232.Si	-0.957078	5.083817	0.485292
233.Si	4.434293	-0.390305	2.246885
234.Si	-2.822531	-0.092122	4.091961
235.U	2.372576	-1.545189	-0.669200
236.U	-2.345415	1.542558	0.756989

Energy: −29169.12 kcal/mol

Table S6. Final coordinates and single point energy of the Z isomer of 3 after geometry optimisation.

1.C	3.485252	3.857613	-6.189382
2.C	2.129142	0.424956	-6.373099

3.C	1.358196	1.415805	-5.476752	54.C	-0.423968	-3.872323	3.276995
4.C	3.851715	3.159901	-4.860593	55.C	3.050419	0.409918	3.389775
5.C	0.027603	0.787526	-5.028142	56.C	-2.690733	-4.787075	3.892116
6.C	-2.894069	-1.834409	-4.954164	57.C	5.915438	2.952248	4.207818
7.C	-4.565101	-0.061421	-4.287681	58.C	2.349986	3.791023	3.984733
8.C	4.540582	4.170037	-3.921280	59.C	-1.651491	-3.685957	4.185386
9.C	-5.894531	-3.647086	-3.748500	60.C	-1.181114	0.802062	4.268403
10.C	0.728716	4.487466	-3.854452	61.C	-0.897129	-0.692618	4.490550
11.C	-3.473006	-1.023391	-3.777734	62.C	3.327013	0.520879	4.906550
12.C	4.892061	-4.135678	-3.353166	63.C	-3.680562	-1.825126	5.618542
13.C	4.208995	0.160538	-3.600279	64.C	-4.056767	-0.398372	6.066511
14.C	5.517844	0.585838	-2.944861	65.C	-0.268570	-0.911841	5.882664
15.C	1.561542	-2.524493	-3.168219	66.C	-3.331985	-2.679484	6.857640
16.C	1.228905	3.293965	-3.014263	67.H	1.525526	0.146293	-7.253579
17.C	-2.573259	-4.519154	-2.659564	68.H	3.098039	3.154669	-6.940769
18.C	1.346104	-4.981056	-2.729845	69.H	4.373803	4.346711	-6.625283
19.C	-5.654468	-2.956802	-2.388380	70.H	3.079123	0.836538	-6.746406
20.C	4.633110	-6.114083	-1.794493	71.H	2.724111	4.640522	-6.047132
21.C	4.682908	-4.574164	-1.887402	72.H	1.112509	2.295260	-6.102430
22.C	-3.152721	2.620929	-2.017663	73.H	2.356548	-0.506800	-5.833024
23.C	1.632188	-3.608929	-2.079664	74.H	-0.532178	0.398786	-5.895830
24.C	-2.513982	-3.232960	-1.805233	75.H	-2.580322	-1.155930	-5.765479
25.C	-2.603394	5.052764	-1.739407	76.H	4.607523	2.394068	-5.116554
26.C	1.764554	3.787562	-1.659826	77.H	-3.629257	-2.532576	-5.383387
27.C	-5.935403	-3.943062	-1.234377	78.H	-4.180498	0.552729	-5.119735
28.C	6.031384	-1.097856	-1.214177	79.H	-5.444386	-0.604557	-4.668989
29.C	-2.522841	3.659889	-1.074162	80.H	0.252558	4.168071	-4.792856
30.C	-5.390915	-0.066244	-0.870163	81.H	-5.818957	-2.943812	-4.590272
31.C	6.011390	1.312183	-0.629285	82.H	5.454694	4.579808	-4.384177
32.C	-5.873816	4.258258	-0.241836	83.H	4.279920	0.321007	-4.687495
33.C	-2.353650	-3.630401	-0.328201	84.H	-2.008804	-2.416180	-4.661715
34.C	4.865723	2.212132	-0.175375	85.H	-0.624250	1.508947	-4.514746
35.C	5.150020	-1.808116	-0.194408	86.H	0.190767	-0.053056	-4.337792
36.C	-6.225608	-0.410701	0.357180	87.H	1.549762	5.172632	-4.115966
37.C	-4.739579	6.166320	0.962455	88.H	4.081381	-4.498964	-4.002650
38.C	-4.864046	4.629797	0.866088	89.H	5.834794	-4.541850	-3.757328
39.C	1.927092	-5.661409	0.699863	90.H	-5.177833	-4.462005	-3.930424
40.C	2.777462	-4.375483	0.712314	91.H	-6.904857	-4.090962	-3.781134
41.C	-1.129582	5.707045	1.405023	92.H	3.882006	5.024126	-3.700274
42.C	3.972265	-4.543369	1.672769	93.H	-2.641611	-4.316293	-3.736170
43.C	3.430305	4.652367	1.864757	94.H	2.367269	-2.630505	-3.908021
44.C	-1.808591	4.406557	1.880081	95.H	-4.910415	0.631224	-3.509476
45.C	-5.679812	1.271899	2.094864	96.H	4.928404	-3.041716	-3.463515
46.C	-4.467918	-2.163570	2.460951	97.H	6.399094	0.119308	-3.423842
47.C	-4.340905	1.881375	2.500096	98.H	0.606657	-2.587143	-3.715874
48.C	-5.589869	-1.144997	2.626125	99.H	4.088052	-0.930069	-3.476238
49.C	2.817232	3.420403	2.562903	100.H	2.094095	-5.221333	-3.499667
50.C	5.729219	2.085165	2.943215	101.H	-0.013184	5.075830	-3.291453
51.C	6.522089	0.768352	3.093865	102.H	-2.648603	-0.397484	-3.384246
52.C	3.362514	-1.024505	2.930689	103.H	4.829344	3.726361	-2.956987
53.C	-2.242611	4.539955	3.354635	104.H	5.619153	1.672894	-3.043379

105.H	0.364496	-4.970272	-3.229485	156.H	3.593760	4.480271	0.792818
106.H	-2.660380	2.645645	-3.003304	157.H	4.683555	-5.303939	1.313353
107.H	5.530002	-6.557205	-2.261965	158.H	2.137952	-3.573316	1.129056
108.H	0.352159	2.656752	-2.796728	159.H	-6.112457	1.878623	1.291366
109.H	-1.672559	-5.130786	-2.492550	160.H	0.516665	0.376363	1.660006
110.H	2.133472	5.023692	-2.735808	161.H	-4.127172	6.482935	1.818691
111.H	3.758199	-6.531428	-2.315623	162.H	-5.309215	4.301875	1.823594
112.H	-3.437587	-5.141320	-2.384783	163.H	-4.547496	-2.619460	1.458119
113.H	1.609088	-1.491632	-2.780986	164.H	1.664856	-5.953487	1.730848
114.H	-6.415934	-2.157322	-2.314826	165.H	4.527347	-3.607859	1.818892
115.H	-4.220486	2.821312	-2.186890	166.H	-0.341581	6.007776	2.116444
116.H	6.030335	-1.681525	-2.142773	167.H	-1.037279	3.612323	1.847102
117.H	1.340769	-5.806198	-2.005881	168.H	4.395217	4.942409	2.310229
118.H	-1.599153	-2.683357	-2.101624	169.H	6.203547	2.647946	2.118311
119.H	-3.645917	5.370194	-1.889577	170.H	2.760253	5.524025	1.955299
120.H	-6.000002	-0.242609	-1.770732	171.H	3.111755	-1.206198	1.875089
121.H	2.648961	4.429924	-1.772707	172.H	1.909760	3.149333	1.988709
122.H	5.588341	-4.264684	-1.331691	173.H	6.410213	0.100814	2.226948
123.H	-3.057556	1.587902	-1.651439	174.H	-6.592117	-1.610521	2.577004
124.H	-6.978119	-4.302516	-1.265165	175.H	-0.673472	-3.692776	2.221152
125.H	-5.288099	-4.831045	-1.295676	176.H	3.624783	-4.872019	2.667076
126.H	-5.568144	4.664920	-1.217681	177.H	-6.400686	1.257097	2.933414
127.H	6.801931	1.878869	-1.155315	178.H	-3.035157	-4.741574	2.847497
128.H	4.599010	-6.466769	-0.753670	179.H	-4.517823	2.849856	2.992687
129.H	0.814099	-3.409557	-1.359988	180.H	7.599463	0.970305	3.220305
130.H	-2.099878	5.836683	-1.159169	181.H	-4.624681	-2.993175	3.168103
131.H	0.999519	4.377819	-1.130114	182.H	4.424009	-1.273594	3.072616
132.H	7.078472	-1.012940	-0.869105	183.H	-0.034535	-4.901424	3.354471
133.H	4.388659	2.642617	-1.074270	184.H	-3.075657	5.250190	3.477352
134.H	2.044065	2.966078	-0.979769	185.H	-3.892452	1.240525	3.280495
135.H	-5.184868	1.018139	-0.863343	186.H	1.965212	0.567706	3.244420
136.H	-1.450387	3.404796	-0.981584	187.H	-5.487775	-0.672089	3.609883
137.H	-5.988052	3.171798	-0.369415	188.H	-1.538101	1.028298	3.250147
138.H	-6.871877	4.672841	-0.019422	189.H	0.397954	-3.190490	3.538043
139.H	-5.770072	-3.491214	-0.245103	190.H	2.779401	-1.753345	3.516591
140.H	-1.495924	-4.311472	-0.201065	191.H	-2.555875	3.578204	3.781170
141.H	-4.295060	6.603761	0.055782	192.H	-1.409028	4.913458	3.973069
142.H	5.611687	-2.776210	0.055452	193.H	5.472187	3.952019	4.097946
143.H	-3.241326	-4.151552	0.056768	194.H	6.196204	0.201273	3.978736
144.H	6.463588	0.847372	0.254263	195.H	-2.250209	-5.787666	4.041118
145.H	2.461064	-6.512233	0.249171	196.H	1.716646	4.693994	3.954671
146.H	-7.192993	0.125262	0.364106	197.H	-0.141157	-0.988411	3.739997
147.H	-0.250056	1.685594	-0.507111	198.H	6.989734	3.091865	4.420974
148.H	-6.438423	-1.486832	0.343659	199.H	1.751613	2.992564	4.446285
149.H	0.982713	-5.529624	0.153519	200.H	-3.577691	-4.721879	4.540549
150.H	5.277298	3.075404	0.370066	201.H	-0.265600	1.398571	4.412140
151.H	-2.155520	-2.781216	0.348977	202.H	3.192101	4.010856	4.659003
152.H	-0.649737	5.589855	0.422739	203.H	5.468502	2.486594	5.099115
153.H	-5.736787	6.623661	1.086145	204.H	-1.933668	1.189951	4.969227
154.H	5.158851	-1.227953	0.744989	205.H	4.390007	0.357147	5.138017
155.H	-1.836900	6.547788	1.334068	206.H	-1.312444	-3.829293	5.229426

207.H	-4.592825	-2.275344	5.184484	223.N	-4.126549	-0.846958	-0.865303
208.H	3.044181	1.495493	5.325193	224.N	3.767112	-1.945658	-0.720543
209.H	-4.313111	0.259365	5.222954	225.N	3.881092	1.432685	0.622316
210.H	2.760431	-0.251397	5.451679	226.N	-3.441567	1.982571	1.319186
211.H	0.033370	-1.956619	6.046137	227.N	-5.442781	-0.101186	1.579988
212.H	0.628974	-0.285441	6.006517	228.N	-3.153815	-1.491089	2.632415
213.H	-3.175844	-3.738513	6.607843	229.Si	2.400189	2.156288	-4.028728
214.H	-4.923504	-0.414490	6.749278	230.Si	-3.961843	-2.021904	-2.195045
215.H	-3.228373	0.080780	6.610680	231.Si	3.234700	-3.621453	-1.009195
216.H	-0.964887	-0.637729	6.689985	232.Si	-3.182791	3.653644	0.738826
217.H	-2.420575	-2.321599	7.361338	233.Si	3.887836	1.816602	2.368375
218.H	-4.149107	-2.634508	7.598674	234.Si	-2.367261	-1.891516	4.177578
219.As	-0.371329	0.312000	-1.192340	235.U	2.778069	0.085060	-0.849926
220.As	0.296852	-0.917245	0.853968	236.U	-2.785701	-0.143084	0.816002
221.N	3.076044	0.891731	-2.974848				
222.N	5.460818	0.243265	-1.500962				

Energy: −29145.08 kcal/mol

References

1. B. M. Gardner, G. Balázs, M. Scheer, F. Tuna, E. J. L. McInnes, J. McMaster, W. Lewis, A. J. Blake, S. T. Liddle, *Angew. Chem. Int. Ed.* **2014**, *53*, 4484.
2. B. M. Gardner, G. Balázs, M. Scheer, F. Tuna, E. J. L. McInnes, J. McMaster, W. Lewis, A. J. Blake, S. T. Liddle, *Nat. Chem.* **2015**, *7*, 582.
3. W. C. Johnson, A. Pechukas, *J. Am. Chem. Soc.* **1937**, *59*, 2068.
4. C. Fonseca Guerra, J. G. Snijders, G. te Velde E. J. Baerends, *Theor. Chem. Acc.* **1998**, *99*, 391.
5. G. te Velde, F. M. Bickelhaupt, S. J. A. van Gisbergen, C. Fonseca Guerra, E. J. Baerends, J. G. Snijders T. Ziegler, *J. Comput. Chem.* **2001**, *22*, 931.
6. S. H. Vosko, L. Wilk, M. Nusair, *Can. J. Phys.* **1980**, *58*, 1200.
7. A. D. Becke, *Phys. Rev. A.* **1988**, *38*, 3098.
8. J. P. Perdew, *Phys. Rev. B.* **1986**, *33*, 8822.
9. S. Portmann, H. P. Luthi, *Chimia* **2000**, *54*, 766.
10. NBO 5.0: E. D. Glendening, J. K. Badenhoop, A. E. Reed, J. E. Carpenter, J. A. Bohmann, C. M. Morales, F. Weinhold, (Theoretical Chemistry Institute, University of Wisconsin, Madison, WI, 2001); http://www.chem.wisc.edu/~nbo5.
